# Supplementary material for: End Sequence Analysis Toolkit (ESAT) expands the extractable information from single-cell RNA-seq data
Source: Genome Res. 2016 Oct;26(10):1397–410. doi: 10.1101/gr.207902.116 (PMC5052061; doi:10.1101/gr.207902.116)
Supplement: Supplemental Material [file supp_gr.207902.116_Supp_Materials.tar.gz › ESAT-esat_v0.1/doc/html/classbroad_1_1pda_1_1seq_1_1clip_1_1_genome_space_sample_data.html]

ESAT: broad.pda.seq.clip.GenomeSpaceSampleData Class Reference


ESAT


- Main Page
- Related Pages
- Packages
- Classes
- Files

- Class List
- Class Index
- Class Hierarchy
- Class Members

All Classes Namespaces Files Functions Variables Enumerator Pages

- broad
- pda
- seq
- clip
- GenomeSpaceSampleData

Public Member Functions |
List of all members

broad.pda.seq.clip.GenomeSpaceSampleData Class Reference

Inheritance diagram for broad.pda.seq.clip.GenomeSpaceSampleData:

[legend]

Collaboration diagram for broad.pda.seq.clip.GenomeSpaceSampleData:

[legend]

|  |  |
| --- | --- |
| Public Member Functions | |
|  | GenomeSpaceSampleData (String bamFile, String chrSizeFile, Map< String, Collection< Gene >> genes, double expressionCutoff, boolean fullyContained) throws IOException |
|  | |
| boolean | isExpressed (Gene gene) |
|  | |
| double | getGenePval (Gene gene) |
|  | |
| Public Member Functions inherited from broad.pda.seq.clip.SampleData | |
|  | SampleData (String bamFile, boolean firstReadTranscriptionStrand, Map< String, Collection< Gene >> genes, double expressionCutoff, boolean expByScanPval, boolean fullyContained) throws IOException |
|  | |
| int | hashCode () |
|  | |
| void | setExpressionScanPvalueCutoff (double expressionScanPvalCutoff) |
|  | |
| double | getExpressionScanPvalueCutoff () |
|  | |
| double | getGeneCount (Gene gene) |
|  | |
| double | getFragmentLengthGeneCount (Gene gene) |
|  | |
| double | getGeneScanPval (Gene gene) |
|  | |
| double | getGeneAverageCoverage (Gene gene) |
|  | |
| boolean | isExpressed (Gene gene) |
|  | |
| boolean | firstReadTranscriptionStrand () |
|  | |
| double | getEnrichmentOverGene (Gene gene, Annotation window) |
|  | |
| Logger | getLogger () |
|  | |
| String | getSampleName () |
|  | |
| String | getOriginalBamFile () |
|  | |
| void | setSampleName (String name) |
|  | |
| TranscriptomeSpaceAlignmentModel | getData () |
|  | |
| TranscriptomeSpaceAlignmentModel | getFragmentLengthFilterData () |
|  | |

|  |  |
| --- | --- |
| Additional Inherited Members | |
| Static Public Attributes inherited from broad.pda.seq.clip.SampleData | |
| static Logger | logger = Logger.getLogger(SampleData.class.getName()) |
|  | |
| Protected Attributes inherited from broad.pda.seq.clip.SampleData | |
| String | sampleName |
|  | |
| TranscriptomeSpaceAlignmentModel | data |
|  | |
| TranscriptomeSpaceAlignmentModel | maxFragmentLengthData |
|  | |
| Map< Gene, ScanStatisticScore > | maxFragmentLengthGeneScores |
|  | |
| Map< Gene, ScanStatisticScore > | geneScores |
|  | |
| Map< Gene, Double > | geneAvgCoverage |
|  | |
| Map< String, Collection< Gene > > | genesByChr |
|  | |
| Map< String, Gene > | genesByName |
|  | |
| double | expressionCutoffValue |
|  | |
| boolean | expressionByScanPval |
|  | |
| boolean | fullyContainedReads |
|  | |

## Detailed Description

Author
:   prussell

## Constructor & Destructor Documentation

|  |  |  |  |
| --- | --- | --- | --- |
| broad.pda.seq.clip.GenomeSpaceSampleData.GenomeSpaceSampleData | ( | String | *bamFile*, |
|  |  | String | *chrSizeFile*, |
|  |  | Map< String, Collection< Gene >> | *genes*, |
|  |  | double | *expressionCutoff*, |
|  |  | boolean | *fullyContained* |
|  | ) |  | throws IOException |

Parameters
:   |  |  |
    | --- | --- |
    | bamFile | Bam alignment file |
    | chrSizeFile | Chromosome size file |
    | genes | Genes by chromosome |
    | window | Window size |
    | step | Step size |
    | expressionCutoff | Genome wide scan P value cutoff for expression |
    | fullyContained | Count fully contained reads only |

Exceptions
:   |  |  |
    | --- | --- |
    | IOException |  |

## Member Function Documentation

|  |  |  |  |  |  |
| --- | --- | --- | --- | --- | --- |
| double broad.pda.seq.clip.GenomeSpaceSampleData.getGenePval | ( | Gene | *gene* | ) |  |

Get genome wide scan P value of number of fragments mapping to the gene Get score from cache or calculate and cache score

Parameters
:   |  |  |
    | --- | --- |
    | gene | The gene |

Returns
:   The scan P value of the number of fragments mapping to the gene

|  |  |  |  |  |  |
| --- | --- | --- | --- | --- | --- |
| boolean broad.pda.seq.clip.GenomeSpaceSampleData.isExpressed | ( | Gene | *gene* | ) |  |

Whether the gene is significantly expressed genome wide

Parameters
:   |  |  |
    | --- | --- |
    | gene | The gene |

Returns
:   Whether the gene is expressed at the given significance level

---

The documentation for this class was generated from the following file:

- java/broad/pda/seq/clip/GenomeSpaceSampleData.java


---

Generated on Fri Aug 15 2014 15:11:01 for ESAT by  

 1.8.7
